# Larvicidal, oviposition inhibition, electrophysiological, histological, and docking activity of *Eugenia uniflora L. oil against Aedes aegypti*

**DOI:** 10.3389/fphar.2026.1688998

**Published:** 2026-06-02

**Authors:** João Vitor Castro Aguiar, Júlio César Ribeiro de Oliveira Farias de Aguiar, Edna Barbosa de Lima, Ana Carla da Silva, Eduarda Florencio Santos, Paulo Gomes Pereira Júnior, Geanne Karla Novaes Santos, Suyana Karolyne Lino da Rocha, José Jorge Almeida de Andrade, Priscila Soares da Silva, Gilson José da Silva Gomes Vieira, Valéria Wanderley Teixeira, Glaucilane dos Santos Cruz, Álvaro Aguiar Coelho Teixeira, Marcílio Martins de Moraes, Cláudio Augusto Gomes da Câmara, Paulo Milet-Pinheiro, André Mesquita Marques, Maria Raquel Figueiredo, Daniela M. A. F. Navarro

**Affiliations:** 1 Chemical Ecology Laboratory, Department of Fundamental Chemistry, Federal University of Pernambuco, Recife, Pernambuco, Brazil; 2 Department of Animal Morphology and Physiology, Federal Rural University of Pernambuco, Recife, Pernambuco, Brazil; 3 Chemistry Department, Federal Rural University of Pernambuco, Recife, Pernambuco, Brazil; 4 Ecology Laboratory, Department of Biological Sciences, University of Pernambuco, Petrolina, Pernambuco, Brazil; 5 Laboratory of Natural Products Chemistry, National Institute for Pantanal Research, Cuiabá, Mato Grosso, Brazil; 6 Department of Natural Products, Farmanguinhos/Fiocruz, Rio de Janeiro, Brazil

**Keywords:** *Aedes aegypti*, curzerene, *Eugenia uniflora*, larvicidal, oviposition deterrence

## Abstract

**Introduction:**

Arboviruses represent a serious problem affecting half of the world’s population, according to the World Health Organization. Natural products are an option for combating this vector.

**Method:**

The essential oil (EO) of *E. uniflora* was extracted by hydrodistillation, while the isolation of the main constituent of the oil (curzerene) was also performed by countercurrent chromatography, with a purity greater than 97%. Larvicidal tests were performed by exposing L4 stage larvae to the EO and the major constituent. The oviposition deterrent effect and the electroantennographic response of *Ae. aegypti* females were also investigated. Histological sections were performed to assess the damage caused by the oil, and docking studies were performed with the major constituent.

**Results:**

Bioassays demonstrated promising larvicidal activity of the essential oil (LC50 = 52.20 ppm) and curzerene (LC50 = 55.70 ppm), as well as a promising oviposition deterrent action, since the oil reduced egg laying by more than 74% at 50 ppm and 65% at 5 ppm, and curzerene reduced egg laying by 65% at 56 ppm. Gas chromatography and electroantennography revealed depolarization of Ae. aegypti antennae by the main constituent of the essential oil (curzerene), which is consistent with the oviposition deterrent activity. Molecular docking studies and histological analyses suggest alterations in the digestive membrane and enzymes during exposure to the essential oil.

**Conclusion:**

Therefore, the essential oil of *Eugenia uniflora* and its main constituent (curzerene) may be options for vector control programs.

## Graphical Abstract

**Figure:**
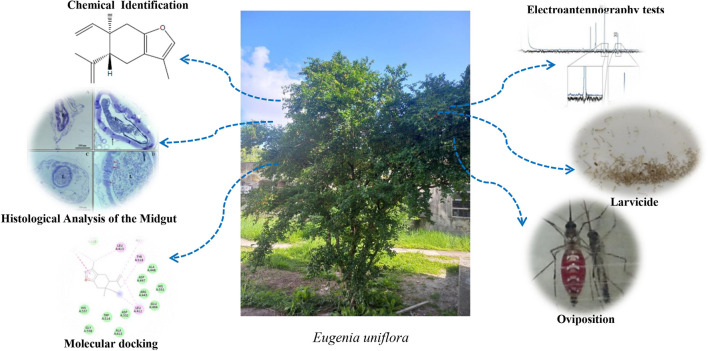

## 1 Introduction

The mosquito *Aedes aegypti* (Linnaeus, 1762) is the vector of the arboviruses that cause dengue, Zika, and chikungunya (Wilder-Smith et al., 2017; Barrera et al., 2019; Leggewie et al., 2023). Due to the considerable dissemination of these diseases by the vector, more than one billion individuals per year are affected, thousands of whom die (Priya et al., 2023). Dengue is widely disseminated and exerts a considerable economic impact throughout the world due to the morbidity and mortality associated with the disease (Torto and Tchouassi, 2024). The high global incidence rates of arboviruses are associated with several factors, such as the increase in human mobility, global warming, and inadequate water storage by urban and rural populations during dry seasons (Colón-González et al., 2018; Salami et al., 2020). These factors increase infection rates by promoting an ideal environment for the proliferation of the vector (MacCormack-Gelles et al., 2020).

Synthetic pesticides and larvicides are widely used to avoid the proliferation of *Ae. aegypti* (Bezerra-Silva et al., 2016). However, the excessive use of these substances has resulted in the emergence of resistant mosquito populations (Kamgang et al., 2020; Kasai et al., 2022). Moreover, the use of pesticides is linked to environmental contamination, as the chemical agents are able to travel through soil, air, and water bodies, affecting non-target organisms and posing risks to human health (Santos et al., 2017; Tudi et al., 2021). Thus, the use of natural products is considered as an efficient, and ecologically safe alternative against the proliferation of mosquitos (Araújo et al., 2020; Dai et al., 2020).

Natural products arising from secondary metabolites of plants have a chemical composition with a vast quantity of bioactive components capable of exerting harmful effect on mosquitos (Warikoo et al., 2011). The plant family Myrtaceae is rich in the production of essential oils and a source of bioactive compounds that can prevent the oviposition of different mosquito vectors (Benelli et al., 2018). The volatile compounds in essential oils (EO) are detected by mosquitos through the olfactory system, which is mainly located in the antennae and maxillary palps, causing behavioral changes in gravid females of *Ae. aegypti*, which could contribute to interrupting the lifecycle of the mosquito (Hill and Ignell, 2021).

The different components of essential oils can present a broad spectrum of bioactivity, with multiple mechanisms of action (Pavela and Benelli, 2016). Studies on essential oils as larvicidal agents for *Ae. aegypti* report important enzymatic effects that cause physiological changes, leading to larval death (Felix et al., 2021; da Silva et al., 2023a). Among species of the family Myrtaceae rich in essential oils, *Eugenia uniflora* stands out for its different chemical profiles (chemotypes), which gives this plant a vast quantity of substances with bioactive potential. Different profiles of this species are reported in the literature. Germacrene B (21.2%) and seline-1,3,7-(11)-trien-8-one oxide (19.3%) were reported as the major compounds in the essential oil of specimens collected in the state of Rio Grande do Sul, Brazil (Vitória et al., 2012). Curzerene was found in specimens collected in the state of Pará (34.4% in April and 53.1% in August) (Da Costa et al., 2020). Germacrone (32.8%), curzerene (30.0%), and germacrene B (15.6%) were described as the major constituents of the oil in other specimens collected in the same state (Maia et al., 1999). Seline-1,3,7(11)-trien-8-one and seline-1,3,7(11)-trien-8-one epoxide were found in specimens collected in the states of Ceará (De Morais et al., 1996) and Rio de Janeiro (Marques et al., 2018).

In this work, we analyzed the chemical composition of the essential oil from *E. uniflora* and its potential as a larvicide and oviposition deterrent. The major constituent of the oil (curzerene) was isolated by countercurrent chromatography and also tested for larvicidal and oviposition deterrent potential. We also performed electroantennography to investigate which compounds of the essential oil from *E. uniflora* are perceived by the antennae of *Ae. aegypti* females.

## 2 Materials and methods

### 2.1 Plant material

Leaves of *E. uniflora* were collected in January 2018 from the campus of the Rural Federal University of Pernambuco (latitude: 08° 01′ 74″ S; longitude: 34° 94′ 92″ W) in the city of Recife, Brazil. The plants were identified by Câmara, C. A. G, and a voucher specimen was deposited in the Professor Vasconcelos Sobrinho Herbarium of the university (voucher n° 48490).

### 2.2 Extraction of essential oil

Fresh leaves from *E. uniflora* were crushed using a blender, and the essential oil was obtained by hydrodistillation in a modified Clevenger apparatus for 3 hours at a temperature of 100 °C. The essential oil obtained was dried using anhydrous sodium sulfate and kept under refrigeration at −4 °C.

### 2.3 Isolation of curzerene by countercurrent chromatography

A countercurrent chromatography system was used (HSCCC model, QuikPrep Ltd., PO Box 80, Bridgend, S. Wales, United Kingdom) composed of a 125-mL stainless-steel coil, high-performance liquid chromatography (HPLC) pump (V10STF01Rev16 series II model, Lab Alliance, United States), low-pressure injection valve (Rheodyne 5020, Cotati, CA, United States), and 5 mL of polytetrafluoroethylene. This system was coupled to a fraction collector (FC203B model, GILSON INC., Middleton, WI, United States) programmed to collect at 1.0 min intervals. The solvent system used for separation was n-hexane/acetonitrile/methanol (5:5:1). The essential oil (700 mg) from *E. uniflora* was dissolved in a biphasic system and injected into the device.

### 2.4 Analytical condition

The components of the *E. uniflora* oil were identified using gas chromatography coupled to mass spectrometry (GC-MS) in an Agilent 5975C chromatograph with a quadrupole detector equipped with an apolar HP-5 column (30 m × 0.25 mm, 0.25 µm, Agilent), with the injector inlet maintained at 250 °C. The initial oven temperature was 40 °C, maintained for 2 min, at a rate of 4 °C/min maintained for 5 min. Helium was the carrier gas, with a flow rate of 1 mL/min. The temperature of the quadrupole detector and ion source was 230 °C and 150 °C, respectively, with an ionization potential of 70 eV and a scanning range of 35–350 m/z at 1.0 scan/s. An aliquot of 1 µL of essential oil solution at 1,000 ppm diluted in acetone was manually injected into the equipment in split mode at 1:50 separately, followed by co-injection with an aliquot of a standard hydrocarbon solution containing a homologous set of alkanes (C_9_ to C_30_). The retention index was calculated for each compound through the analysis of the retention time of each component of the sample and the hydrocarbon standards, using the equation proposed by Van den Dool and Kratz (1963). The compounds were identified by comparing the calculated retention indices to standard indices available in commercial libraries (Adams, 2007) and comparisons of the mass spectra obtained experimentally to mass spectra of authentic standards. The quantification was performed using GC- FID (Thermo trace GC ultra) with a VB-5 column (60 m × 0.25 mm; film thickness: 0.25 μm), with nitrogen as the mobile phase (carrier gas) and temperature conditions the same as those reported for GC-MS (da Silva et al., 2023a). The relative percentage of each component of the sample was determined based on the areas of the peaks in the chromatograms.

### 2.5 Electrophysiological analysis

This technique was performed exploratory to investigate potential candidates for oviposition activity present in the essential oil whose deterrent behavior could be experimentally verified. This analysis was conducted to investigate which potential compounds in the essential oil stimulated the antenna of the female mosquitos. Tests were performed in a gas chromatograph with a flame ionization detector (Thermo Trace GC Ultra, Thermo Scientific, Milan, Italy) coupled to an electroantennographic detector (Syntech, Kirchzarten, Germany) (GC-EAD). The gas chromatograph was equipped with an HP-5 apolar column (50 m × 0.25 mm x 0.25 µm, Agilent). Nitrogen was the carrier gas and kept at a constant flow rate of 1 mL/min. The initial oven temperature was 100 °C, maintained for 1 min, ramped up to 210 °C at a rate of 7 °C/min, and held for 2 min. The injector and detector temperatures were both 250 °C and the transference line for the EAD was heated to 220 °C. The coupling of the GC-EAD was composed of a temperature control module of the transference line (TC-02), a stimulus control module (CS-55), a module to control the acquisition of data captured by the electrodes (IDAC4), and a flow divider to enable the components exiting the chromatographic column to be directed simultaneously (through deactivated columns) to both the FID and a cylindrical glass tube through which clean, humidified air passed, which directed the separated compounds to the antennae of the mosquito. Females mosquitoes between two and 7 days were used for the electroantennographic analysis after fasting for 24 h. The mosquitos were put into a state of dormancy through refrigeration (4 °C for 10–20 min), followed by decapitation with a scalpel. The head was placed in a capillary tube containing Ringer’s solution and the distal tip of one of the antennae was cut. The tube containing the head of the mosquito was inserted into one of the electrodes of the EAD and the cut tip of the antenna was connected to a capillary tube in the second electrode, thus closing the circuit. The antenna between the two tubes was then placed at the opening of the cylindrical tube through which the effluents of the GC-EAD passed. The antennae of *Ae. aegypti* females are more difficult to maintain stable electrophysiological responses to GC-EAD signals compared to those of other insects, but after assembling several antennae, it was possible to achieve the desired stability to the point where a trend in the responses was observed. These data were obtained after 10 repetitions performed with 10 different mosquito antennae under the same conditions. However, only compounds detected by FID for which a response was observed through a deviation from the EAG baseline above the signal-to-noise ratio and in at least five individuals were considered electrophysiologically active. For these experiments, solutions of the *E. uniflora* oil were prepared at a concentration of 6,000 ppm, using acetone as the solvent.

### 2.6 Biological tests

#### 2.6.1 Mosquitos

*Ae. aegypti* mosquitos (Rockefeller strain) were obtained from the colony of the Chemical Ecology Laboratory of the Department of Fundamental Chemistry of the Federal University of Pernambuco in the city of Recife, Brazil. The mosquitos were kept in a controlled climate with a temperature of 27 °C ± 1 °C, 70% ± 5% relative humidity, and 14-h photoperiod and fed a 10% sucrose solution (Supplementary material S1).

#### 2.6.2 Larvicidal bioassay

The larvicidal tests were performed based on the method recommended by the World Health Organization modified by Navarro et al. (2003). Separate stock solutions were prepared with the essential oil and the isolated compound curzerene at 100 ppm using Tween 80 as the solvent and distilled water. The larvicidal tests were performed using these solutions at essential oil or curzerene concentrations of 50, 60, 70, 80, 90, and 100 ppm. For the bioassay, 20 larvae (L_4_) were distributed in glass containers with a final volume of 20 mL. All bioassays were performed in triplicate, along with the positive control (Temefos at 1.0 ppm) and negative control (distilled water and Tween 80). Mortality was determined after 48 h of exposure to the solutions (Supplementary Material S2 and S3).

#### 2.6.3 Oviposition deterrence test

For the oviposition deterrence bioassays, solutions were prepared based on the lethal concentrations (LC_50_) found in the larvicidal tests. The essential oil was used at a concentration of 50 ppm, which was subsequently diminished to 5 ppm. Curzerene was used at a concentration of 56 ppm. For the oviposition experiments, 25 mL of the test solutions and control solutions (distilled water and ethanol) were placed in glass containers containing filter paper measuring 12 × 12 cm (Supplementary material S4). The females were fed a blood meal, and oviposition assays were performed on the fourth day after feeding. The glasses were distributed randomly in eight metal and acrylic cages (33 × 21 × 30 cm) covered with fabric, with 10 gravid females in each cage (Supplementary material S5). The females were left in the dark for 16 h (Supplementary material S6), followed by counts of the number of eggs laid on the filter paper in each glass (Supplementary material S7) to determine the oviposition activity index (OAI) (Supplementary material S8). A blank test was also conducted with ethanol and distilled water versus ethanol and distilled water to assess possible external interferences.

### 2.7 Statistical analyses

The lethal concentration for 50% of the larvae (LC_50_) was calculated using Probit analysis and a 95% confidence interval with the aid of the Statplus® 2009 software. Larvae not rising to the surface and with an absence of movement after 48 h of exposure to the solutions were considered dead. The oviposition tests were analyzed considering the oviposition activity index (OAI), according to Kramer and Mulla (1979). The paired Student’s t-test was used to determine significant differences in means with the aid of the Minitab® 19 software, considering a 5% significance level. (Supplementary material: Statistical Analysis of Larvicidal and Oviposition Bioassays).

### 2.8 Histological analysis of the midgut

The midgut of surviving larvae was collected at the established time points (12 and 24 h) and fixed in 10% buffered formaldehyde for 24 h. Subsequently, it was dehydrated and processed for embedding in historesin (Leica, Solms, Germany). Finally, the tissue was observed under an optical microscope (Olympus BX60, Olympus America, Inc., NY, United States) and photographed with a digital camera attached to the microscope. Five larvae were used per treatment (de Almeida et al., 2021).

### 2.9 Molecular docking

In silico molecular docking studies were performed using *Ae*. *aegypti* α-amylase (UniProt ID: P53354) as the receptor, a key enzyme in the carbohydrate metabolism of the insect and a strategic molecular target for vector control (da Silva et al., 2023a).

The three-dimensional structures of the ligands Curzerene (PubChem CID: 165365640), Selina-1,3,7(11)-trien-8-one (PubChem CID: 91746501), and Oxidoselina-1,3,7(11)-trien-8-one (PubChem CID: 91750195) were obtained from the PubChem database.

Molecular docking simulations were carried out using the DockThor-VS server, employing MMFF94S force field, 24 independent runs, 1,000,000 evaluations per run, and a population size of 750 individuals. This protocol follows a previously validated methodology for virtual screening and molecular docking investigations (Guedes et al., 2024).

The grid box was defined based on residues reported as relevant for interactions with sesquiterpenes da Silva et al. (2023a), ensuring full coverage of the catalytic cavity of the protein. The geometric center of the grid was established using residues TRP315, TYR318, GLN319, and LEU415, with coordinates X = 7.1379, Y = −5.6038, and Z = −15.5422, and dimensions of 6.058 Å. This region corresponds to the catalytic site responsible for polysaccharide hydrolysis and for interactions with natural enzyme inhibitors in *Ae*. *aegypti* (da Silva et al., 2023a).

The best binding poses were selected based on the binding energy calculated using the DockTScore scoring function and were subsequently analyzed using BIOVIA Discovery Studio Visualizer 2025.

## 3 Results

### 3.1 Chemical composition of essential oil from leaves of *Eugenia uniflora*

The yield of oil was 1.02%. The gas chromatography/mass spectrometry (GC/MS) (Supplementary material S8) and gas chromatography/flame ionization detection (GC/FID) analyses enabled the identification of 21 compounds, which accounted for 91.68% of the essential oil (Table 1). The identified constituents were sesquiterpenes, such as (*E*)-Caryophyllene (16.68%), β-Elemene (12.67%), Germacrene D (4.97%), δ-Elemene (4.19%), and oxygenated sesquiterpenes, such as Curzerene (39.40%), Atractylone (1.08%) and germacrone (0.81%). The major compound was curzerene (39.40%), which was separated by countercurrent chromatography, with 97.63% purity.

**TABLE 1:** Chemical composition of essential oil from leaves of *Eugenia uniflora using GC-MS and GC-FID*.

Compound	RI cal^a^	RI lit^b^	%	SD^c^
Linalool	1097	1095	0.80	0.10
δ-Elemene	1335	1335	4.19	0.14
β-Elemene	1389	1389	12.67	0.33
(E)-Caryophyllene	1416	1417	16.68	0.64
γ-Elemene	1430	1434	0.01	0.01
α-Humulene	1450	1452	0.55	0.45
9-epi-(E)-Caryophyllene	1457	1464	0.72	0.54
Germacrene D	1478	1480	4.97	0.45
β-Selinene	1483	1489	0.01	0.01
Amorpha-4,7(11)-diene	1488	1479	0.01	0.01
**Curzerene**	**1496**	**1499**	**39.40**	**1.05**
Germacrene A	1502	1508	1.48	0.58
δ-Cadinene	1520	1522	0.28	0.01
Selina-3,7(11)-diene	1539	1545	0.64	0.10
Germacrene B	1555	1559	6.25	0.51
Spathulenol	1575	1577	0.11	0.02
trans-β-Elemenone	1601	1602	0.33	0.08
epi-α-Cadinol	1638	1638	0.48	0.21
neo-Intermedeol	1651	1658	0.21	0.03
Atractylone	1658	1657	1.08	0.15
Germacrone	1692	1693	0.81	0.38
Total identified (%)	​	​	91.68	​

^a^ Calculated retention index based on homologous series of n-alkanes.

^b^ Retention index from literature (Adams, 2007).

^c^ Standard deviation of peaks integrated by GC-FID.

^c^ The bold values indicates major component.

### 3.2 Larvicidal activity

Cheng et al. (2003) described the larvicidal potential of essential oils based on LC_50_ values, with LC_50_ < 50 ppm indicating a highly active oil, <100 ppm indicating an active oil, and >100 ppm indicating an inactive oil. Based on these criteria, the essential oil from the leaves of *E. uniflora* (LC_50_ = 52.20 ppm) and its major compound (curzerene) (LC_50_ = 55.70 ppm) are considered active and therefore have larvicidal potential for fourth instar larvae of *Aedes aegypti*, as shown in Table 2. The positive control (Temefos) achieved 100% mortality, whereas no mortality occurred in the presence of the negative control (distilled water and Tween 80).

**TABLE 2:** Larvicidal activity of EO from *Eugenia uniflora* and isolated compound (curzerene) against *Ae. aegypti*.

Substance	N^a^	DF^b^	*x* ^2^	Slope (±SE)	LC_50_ (CI 95%)^c^ ^,^ ^d^ (LLC-ULC)^e^	LC_90_ (CI 95%)^c^ ^,^ ^d^ (LLC-ULC)^e^
EO	720	4	0.781	4.20 ± 0,50	52.2 ± 1.0 ppm (47.0–56.1)	105.0 ± 1.1 ppm (95.8–122.0)
Curzerene	340	4	0.764	5.15 ± 0,77	55.7 ± 1.0 ppm (49.6–60.1)	98.8 ± 1.1 ppm (89.2–117.0)

^a^ Number of larvae used in test.

^b^ Degrees of freedom.

^c^ Lethal concentration and confidence interval.

^d^ Calculated using StartPlus.

^e^ Estimated lower and upper lethal concentration. Negative control: Tween 80 and distilled water (0% mortality); Positive control: temefos at 1 ppm (100% mortality).

### 3.3 Electroantennography tests

**FIGURE 1:**
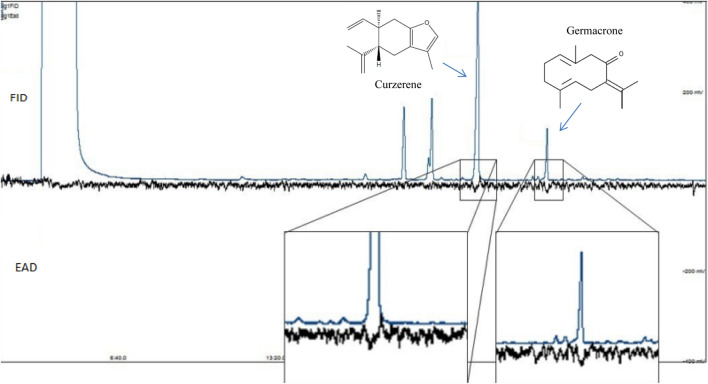
Electrophysiological response of *Aedes aegypti* female antennae to compounds present in the essential oil of *Eugenia uniflora* with simultaneous detection by FID (signal in the upper line in blue) and electroantennographic detection (signal in the lower line in black). In the graph, the horizontal axis represents the chromatographic run time in seconds, while the vertical axis represents the amplitude of the voltage fluctuation of the mosquito antenna in mV.

To determine specific volatile compounds in the complex matrix of the essential oil from *E. uniflora* responsible for the oviposition deterrent effect against *Ae. aegypti*, were combined gas chromatography and electroantennography (CG-EAD) with oviposition bioassays. GC-EAD using the *E. uniflora* essential oil suggested that two compounds (curzerene (Wilder-Smith et al., 2017) and germacrone (Barrera et al., 2019)) may have caused depolarization in the antennae of the *Ae. aegypti* females, one of which was the major constituent of the EO–curzerene (Wilder-Smith et al., 2017) (Figure 1). Curzerene was the major compound (39.40%) of the chromatographic profile of the sample, whose retention time was 28.583 min. This compound had the greatest intensity in the FID.

### 3.4 Oviposition deterrent activity

The EO from *E. uniflora* demonstrated oviposition deterrent potential for *Ae. aegypti* females at a concentration of 50 ppm, with 74% deterrence (p < 0.001) and an oviposition activity index (OAI) of −0.47. The deterrent rate remained high when the EO was diluted to 5 ppm, with 65% deterrence (p < 0.001) and an OAI of −0.31. The isolated compound curzerene at a concentration of 56 ppm achieved 65% deterrence (p < 0.001), with an OAI of −0.31 (Table 3).

**TABLE 3:** Percentage of deterrence and oviposition activity index (OAI).

Substance	OAI	Control (%)	Test (%)	Total eggs	p
Water-ethanol × water-ethanol (blank)	0.08	46	54	3,837	0.665
Essential oil (50 ppm)	−0.47	74	26	2,915	<0.001
Essential oil (5 ppm)	−0.31	65	35	6,644	<0.001
Curzerene (56 ppm)	−0.31	65	35	4,778	<0.001

**FIGURE 2:**
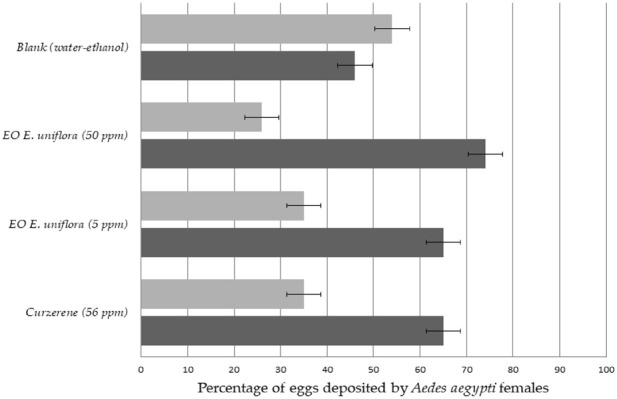
Oviposition response of gravid *Ae. aegypti* females to *Eugenia uniflora* essential oil with respective standard deviations (SD = ±3.7).

According to Kramer and Mulla (1979), OAI values are range from +1 to −1 range, with positive values indicating a preference for the test substance (attractant) and negative values indicating a preference for the control substance (deterrent). Therefore, the results displayed in Table 3 show the deterrent potential of the EO and cruzerene. Figure 2 shows the oviposition results for the EO and its major constituent (curzerene) in percentage terms and total number of eggs in each test.

### 3.5 Histological analysis of the midgut

**FIGURE 3:**
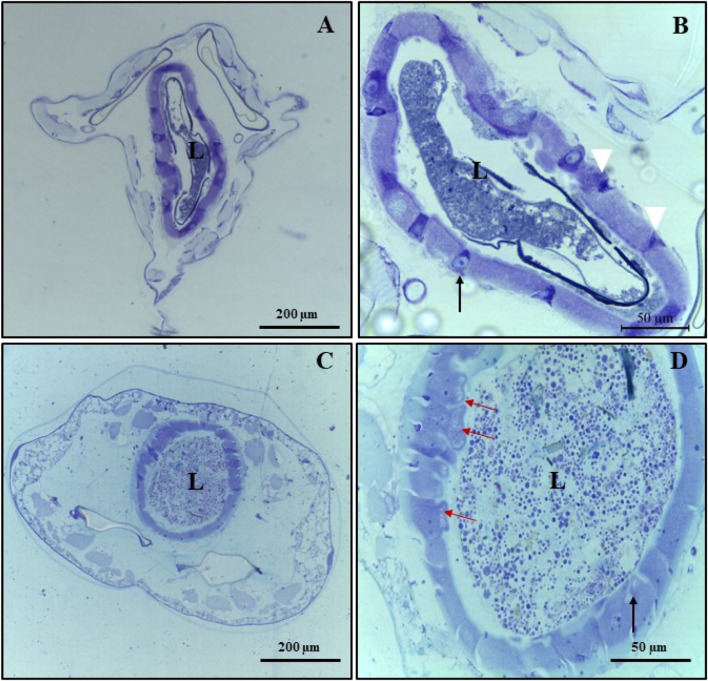
Photomicrograph of the midgut of 4^o^ instar larvae of *Aedes Aegypti*. **(A)** e **(B)** control, 10x e 40x. **(C)** e **(D)** treatment with *Eugenia uniflora* essential oil at LC_50_ (52.2 ± 1.0 ppm), 10x e 40x. L: lumen; black arrow: nucleus; red arrow: cellular protuberance and vacules; white arrowhead: nest of regenerative cells. Azul de Toluidina.

The larvicidal activity of *E. uniflora* EO in LC50 stimulated the investigation of its effects on the morphology and physiology of the L4 midgut. In this sense, histological analyses of larvae from the control group were performed (Figure 3A,B), where the presence of a preserved intestinal epithelium around the lumen (L) can be observed. The nuclei can be seen (red arrows). It is possible to identify the lumen (L), which was well defined and surrounded by a peritrophic matrix secreted by the columnar epithelial cells of the intestine. These cells have a central nucleus and at their apex, microvilli can be seen, forming a brush border with the aim of increasing the absorption surface. Furthermore, regenerative cells with a pyramidal shape were found at the base of the epithelium, aiming at cell replacement. While in Figure 3C it was also possible to observe cellular alterations marked by the presence of protuberances in the apical region and cytoplasmic vacuolization. It is possible to visualize the formation of secretory vesicles along the entire length of the epithelium (apocrine secretion) being expelled towards the intestinal lumen after treatment with the essential oil (red arrows, Figure 3D). It should also be remembered that even without observing cell rupture, essential oils have the property of altering the permeability of the lipid membrane.

### 3.6 Molecular docking

**FIGURE 4:**
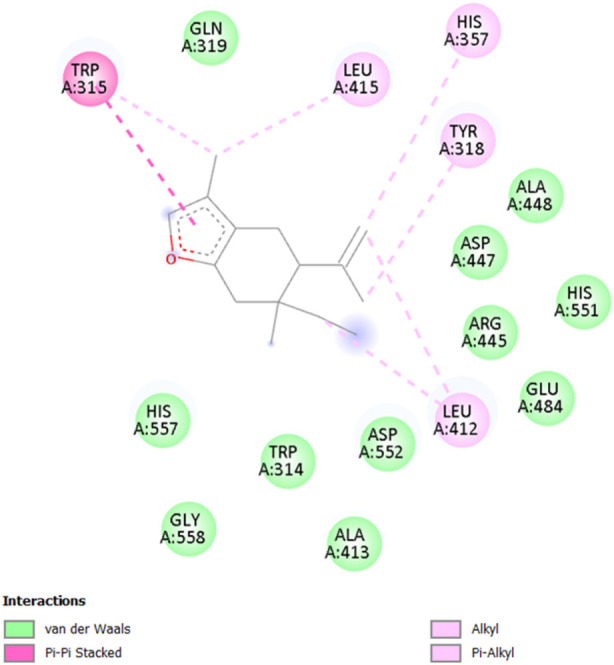
Interactions of curzerene at the active site.

Curzerene exhibited an interaction pattern predominantly governed by hydrophobic and π-alkyl interactions, mainly involving residues LEU412, LEU415, TYR318, HIS357, HIS451, LYS450, ASP447, GLU484, and ALA448, indicating a deep accommodation within the catalytic site (Figure 4). This interaction profile is consistent with the apolar nature of the ligand and suggests that its affinity for the enzyme is primarily driven by van der Waals forces and dispersion interactions, a behavior frequently observed for sesquiterpenes interacting with enzymatic targets (Guedes et al., 2024).

**FIGURE 5:**
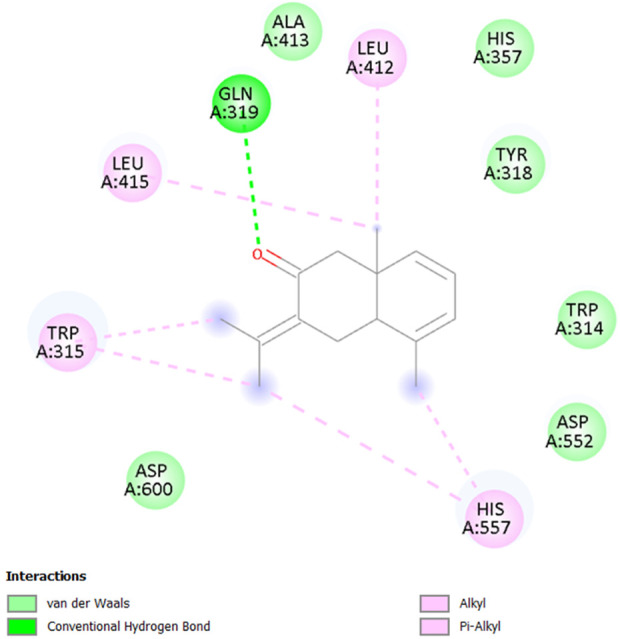
Iterations of Selina-1,3,7(11)-trien-8-one at the active site.

Selina-1,3,7(11)-trien-8-one displayed a predominantly hydrophobic interaction profile, establishing contacts with residues such as LEU412, TYR318, HIS451, HIS557, and TYR401 (Figure 5). Although it presented a lower number of interactions compared to the other ligands, its anchoring within the active site reinforces the importance of the hydrophobic microenvironment of α-amylase in stabilizing terpenoid compounds, as previously described in in silico studies involving natural metabolites (da Rocha et al., 2026).

**FIGURE 6:**
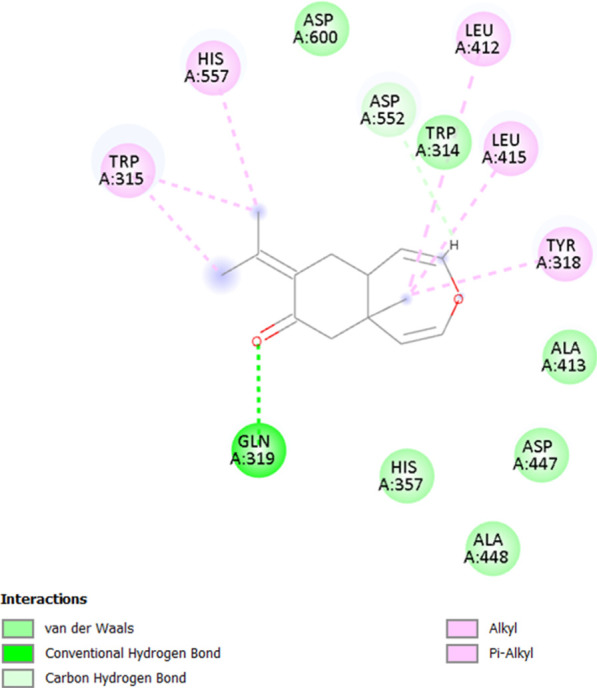
Iterations of Oxidoselin-1,3,7(11)-trien-8-one at the active site.

In contrast, Oxidoselina-1,3,7(11)-trien-8-one stood out by exhibiting, in addition to multiple hydrophobic and π-alkyl interactions with TRP314, TRP315, TYR318, LEU412, and LEU415, a hydrogen bond with residue GLN319, recognized as a key amino acid in the catalytic site of α-amylase (Figure 6). The presence of this additional polar interaction may contribute to the greater stability of the complex and explains the improved binding affinity observed for this ligand, in agreement with previous reports highlighting the importance of GLN319 in anchoring natural enzyme inhibitors (da Silva et al., 2023a).

Overall, the results indicate that the three sesquiterpenes share a core set of active-site residues, particularly TRP314, TYR318, LEU412, and LEU415, which define the hydrophobic microenvironment responsible for molecular recognition by α-amylase. This interaction pattern suggests that the ligands compete with the natural substrate of the enzyme for the same functional domain, supporting the hypothesis of competitive inhibition and corroborating previous findings on the role of terpenoid compounds as modulators of digestive activity in *Ae*. *aegypti* (da Silva et al., 2023a; da Rocha et al., 2026).

## 4 Discussion

### 4.1 Chemical composition of essential oil from leaves of *Eugenia uniflora*

The chemical composition of the essential oil from *E*. *uniflora* has a considerable diversity of compounds. In this study, the major compounds were curzerene (39.40%) and (*E*)-Caryophyllene (16.68%). Different studies describe other findings for the essential oil from this species. The major compounds of the essential oil analyzed by da Cunha et al. (2015) were curzerene, γ-elemene, and atractilone, whereas the major volatile substances described for this oil by Vitória et al. (2012) were germacrene B, selina-1,3,7-trien-8-one, β-caryophyllene, germacrene A, germacrene D, selina-1,3,7-trien-8-one, and curzerene. The essential oil was extracted from the leaves of *E. uniflora* through hydrodistillation in both studies. In the study conducted by Rodrigues et al. (2013), the major constituents were curzerene, γ-elemene, and trans-β-elemenone. Thus, the chemical composition of the essential oil extracted in the present investigation is more similar to that described by da Cunha et al. (2015) and Rodrigues et al. (2013), who report curzerene to be one of the most abundant compounds in the *E. uniflora* oil. Thus, this study is relevant in describing the presence of another chemotype for this plant in northeastern Brazil. da Silva et al. (2023a) showed the presence of selina-1,3,7(11)-trien-8-one and selina-1,3,7(11)-trien-8-one epoxide. These EOs were extracted in the northeast, but in different locations, which shows the influence of geographic location and environmental conditions on the presence of different chemotypes.

### 4.2 Larvicidal activity

Different studies reported biological activities for the *E. uniflora* and demonstrated that extracts of this plant are efficient larvicidal agents for combating *Ae. aegypti* (de Almeida et al., 2021). The authors found that the methanolic extract from the leaves of *E. uniflora* had LC_50_ values of 2.74 and 2.18 mg mL^−1^ at exposure times of 24 and 48 h, respectively, for fourth instar *Ae. aegypti* larvae. The essential oil, aqueous extract, and hydrolate from *E*. *uniflora*, whose major constituents were selina-1,3,7(11)-trien-8-one and selina-1,3,7(11)-trien-8-one epoxide, exhibited promising larvicidal results (da Silva et al., 2023a).

The genus *Eugenia* is recognized for its significant efficacy against insect vectors of the dengue, zika, and chikungunya arboviruses. Da Silva et al. (2015a) described the larvicidal activity of the essential oil from *Eugenia brejoensis* in bioassays involving fourth instar larvae of *Ae. aegypti*, reporting LC_50_ = 214.7 ppm. Raghavendra et al. (2013) analyzed leaf extracts from *Eugenia jambolana* and found LC_50_ = 40.97 and LC_90_ = 83.29 ppm for *Ae. aegypti*. Silva et al. (2020) tested extracts from *Eugenia calycina* for combating third instar larvae of *Ae. aegypti*, reporting LC_50_ = 199.3 and LC_50_ = 166.4 ppm after 24 and 48 h of exposure, respectively. Therefore, the larvicidal results in this study for the EO from *E. uniflora* and its isolated compound curzerene support the bioactive potential described for the genus.

Furthermore, this study uses electrophysiological detection as an exploratory technique for some components of *E*. uniflora essential oil, combined with behavioral oviposition tests as potential compounds detected by the antennae of female mosquitoes. The larvicidal and oviposition tests in this study were performed using a different chemotypic profile than that described in the literature for *E*. uniflora oil, whose main constituents are different (selin-1,3,7(11)-trien-8-one and selin-1,3,7(11)-trien-8-one epoxide), as described by da Silva et al. (2023a). This study also uses a different methodology from the larvicidal study by Spinozzi et al. (2025), which uses dimethyl sulfoxide (DMSO) as a solvent in a ratio of 1:10 (V/V) for myrrh gum resin essential oil and 1:20 for cuzerene. Therefore, the larvicidal tests used in these studies use distilled water as a solvent and to aid in the dissolution of the oil, a surfactant, tween 80, which is less toxic than DMSO (density = 1.10 g.mL^−1^) is used. The mass of the co-solvent (surfactant) used was approximately three drops of tween 80 (density = 1.06 g.mL^−1^), which is equivalent to a mass of 2.6 mg for a 50 mL volumetric flask, which corresponds to a concentration of 52ppm or 0.24% (V/V), which is too low to cause mortality as observed experimentally in the negative control or even described in the literature by Kramer et al. (1983) for the toxicity of Tween 80 (LC50 = 8%, v, v).

Curzerene is an oxygenated sesquiterpene described in the literature for its larvicidal action against several mosquitoes such as *Anopheles subpictus, Aedes albopictus*, and *Culex tritaeniorhynchus* (Govindarajan et al., 2017). Studies also highlight that sesquiterpenes have greater larvicidal activity than monoterpenes against *Ae. aegypti* mosquito larvae, due to their increased lipophilic properties, resulting in increased transmembrane absorption in the body, which can cause toxic effects such as structural alterations of the membranes (Simas et al., 2004). Thus, cuzerene (LC50 = 55.7 µL/1.0 ppm) and the essential oil (LC50 = 52.2 µL/1.0 ppm) of *E*. *uniflora* demonstrated similar LC50 values, therefore, both can be considered active. However, this oil and cruzeren itself can be combined with other oils or isolated compounds (the commercial product such as temephos, for example) to develop larvicidal and oviposition deterrent formulations, so that their synergistic action can be an ecologically renewable alternative that acts on several mechanisms of action simultaneously, enhancing the insecticidal action through different pathways, instead of a single pathway, as is the case with the application of only one larvicidal agent (Sarma et al., 2019).

This work, therefore, contributes to an ecological and renewable alternative for larval control through the larvicidal action of *E*. *uniflora* essential oil (EO) and its major constituent (curzerene). This plant is therefore a sustainable source for extracting essential oils with larvicidal and oviposition-promoting properties, especially in a context of growing insect resistance to synthetic pesticides. The action of this EO and curzerene can be enhanced with the aid of nanotechnology, allowing for greater solubility in nonpolar solvents and improving the bioavailability of bioactive compounds, enabling their application on various types of materials and surfaces that will serve as barriers to the proliferation of mosquitoes and *Ae*. *aegypti* larvae.

### 4.3 Electroantennography tests

Gas Chromatography-Electroantenographic Detection (GC-EAD) analysis was designed purely as a qualitative and exploratory screening tool, with the primary goal of identifying potential biologically active compounds in the essential oil blend. This qualitative screening served as the basis for selecting specific compounds for further evaluation in subsequent behavioral bioassays, which represent the final validation of compound activity in any chemical ecology studies. Thus, despite the subtle amplitude electrophysiological response of curzene, the consistent detection of a low-intensity signal, even observed repeatedly on the antennae of different mosquitoes, is sufficient to indicate a specific interaction with antennal receptors, making it a valid candidate for future behavioral investigations.

Conducting additional GC-EAD analyses with synthetic mixtures and the use of multiple GC columns are excellent strategies for robust validation. However, these strategies are not universally required for exploratory GC-EAD studies, and many highly reputable publications employ GC-EAD only for initial screening with original samples (see, for example: da Silva et al. (2015b), Milet-Pinheiro et al. (2014), Heiduk et al. (2015), Santos et al., (2021)). Furthermore, practical challenges significantly limited our ability to prepare a synthetic mixture of curzene and germacrone in a ratio similar to that found in the essential oil and expose female mosquito antennae in a GC-EAD assay to confirm the activity of the compound(s). Germacrone is present in very low quantities and difficult to isolate, making the preparation of the mixture complex and costly for performing multiple replicates of the electrophysiological assays. Although curzene was isolated, its quantity was adequate only for initial assays, not for extensive testing of synthetic mixtures. To identify the compounds, we used a combination of GC-EAD and GC-MS, comparing retention times and mass spectra with databases (e.g., NIST and Adams) and authentic standards (for curzene), which provided sufficient confidence for this exploratory work.

Thus, these practical limitations and the qualitative and exploratory nature of our study led us to prioritize validation through behavioral assays. However, other essential oils that allow the isolation of sufficient quantities of germacrone to enable various electrophysiological assays will be considered for our future work on the chemical ecology of mosquitoes.

This is, to the best of our knowledge, the first report on the stimulatory effect of curzerene on the antennae of *Ae. aegypti* females. Thus, there is currently no research available in the literature that demonstrate the electrophysiological response to curzerene through behavioral tests. Our results show that curzerene is capable of affecting the oviposition activity of female mosquitoes at the experimental concentrations tested, as the females were able to detect the presence of this compound upon landing at the egg-laying site. This result together with the significant deterrent activity of curzerene found in the oviposition tests (Figure 1), suggests that this substance would be responsible for the biological activity against the mosquito vector. In this study, as it was only possible to verify the oviposition activity for curzerene, this supports the idea that this compound is actually detected in the EAD tests.

Other compounds found in this essential oil also exhibited activity in the electroantennographic test, such as (*E*)-caryophyllene and germacrene D. Previous studies have reported that some of these compounds are able to cause the depolarization of the antennae of *Ae. aegypti* females and cause oviposition deterrence (Santos et al., 2021). Campbell et al. (2011) reported strong responses to (*E*)-caryophyllene and germacrene D in electroantennographic tests. Moreover, other compounds present in the essential oils of plants were capable of depolarizing the antennae of *Aedes* females, such as n-decanol, 2-undecanone, undecanal, dodecanal, trans-caryophyllene, (*E*)-β-farnesene, α-humulene, n-dodecanol, isodaucene, and dodecanoic acid, with n-dodecanol and dodecanal exhibiting strong oviposition deterrence (Bezerra-Silva et al., 2016). Therefore, GC-EAD is highly efficient at suggesting possible natural candidates to serve as oviposition deterrents.

### 4.4 Oviposition deterrent activity

Benelli et al. (2018) investigated oviposition deterrence for *Ae. aegypti* using the essential oil from the leaves of *Syzygium lanceolatum* (Myrtaceae) at concentrations ranging from 50 to 250 ppm, reporting 91.64% deterrence at 250 ppm and 74.68% at 50 ppm, the latter of which is similar to the rate found in this study. The chemical composition of Syzygium EO presents as major compounds: Phenylpropanal (18.3%), α-Humulene (14.5%), (*E*)-Caryophyllene (12.8%) and Caryophylleneoxide (10.7%). The common compounds with this study are: δ-Elemene (1.4%), β-Elemene (5.9%), (*E*)-Caryophyllene (12.8%) and Selinene β (3.4%). Other studies have demonstrated the influence of essential oils on the behavior of female mosquitos. da Silva et al. (2016) analyzed the effects of the essential oil from leaves of *Piper corcovadensis* (Piperaceae) and found more than 50% reduction in the number of eggs laid, with 87.7% and 61.9% deterrence rates at concentrations of 50 and 5 ppm, respectively. da Silva et al. (2023b) found significant oviposition deterrent activity for the essential oil from *E*. *uniflora* and its major compounds (selina-1,3,7(11)-trien-8-one and selina-1,3,7(11)-trien-8-one epoxide) as well as the aqueous extract and hydrolate (byproducts of hydrodistillation). Thus, the present findings are in agreement with deterrence results previously described for the same species and show of its bioactive potential even with a different chemical composition. Gonzalez-Castro et al. (2024) investigated the activity of the ethanolic extract of *Laurencia johnstonii* and its major constituent (laurinterol) against *Ae. aegypti*, reporting a deterrence rate of 79.2%. Santos et al. (2012) found that the essential oil from *Syagrus coronata* (Arecaceae) at a concentration of 50 ppm had an OAI of –0.35, indicating a deterrent effect. Comparing findings literature toour results, one can state that the essential oil from *E. uniflora* exhibited promising results, with a more than 65% reduction in the number of eggs laid by *Ae. aegypti* at a concentration of 5 ppm, which is a significantly lower concentration than that described as having deterrent activity in the majority of studies.

### 4.5 Histological analysis of the midgut

As expected, the larval peritrophic matrix was intact. The presence of secretory vesicles along the entire length of the epithelium (apocrine secretion) being expelled towards the intestinal lumen can be visualized. The formation of secretory vesicles in the apical portion of the epithelial cells of the larval intestine may indicate that the cytoplasmic content was being excited into the intestinal lumen in an attempt to defend the larva’s body against toxic compounds (Valotto et al., 2011).

The peritrophic membrane prevents contact between the intestinal contents and the epithelial cells, enveloping the food bolus (Napoleão et al., 2018). It is worth noting that it has selective permeability and, in this sense, toxic components of the EO may have crossed the peritrophic membrane at some point along its length and stimulated the larval intestine epithelium to respond with cellular alteration and vesicle secretion. Another possibility is that the retention of EO components in the endoperitrophic space may have stimulated these reactions.

In this way, the larvicidal activity of *E. uniflora* EO may be related to alterations in the digestive membrane, such as the inhibition of digestive proteases, which caused damage to the morphophysiology of the larval midgut and induction of dysbiosis. It is known that nonpolar compounds present in the constituents of EO can accumulate in the adipose tissues of living organisms due to their slow partitioning into lipids, resulting in their cellular disorganization over the exposure time.

### 4.6 Molecular docking

Molecular docking studies performed on the DockThor-VS server indicated that the sesquiterpenes Curzerene, Selina-1,3,7(11)-trien-8-one and Oxidoselina-1,3,7(11)-trien-8-one exhibit favorable affinity for *Ae*. *aegypti* α-amylase, with affinity values (docking score) of −8.870, −8.644 and −8.880, respectively (Table 4). These results suggest an indication of a favorable interaction of stable complexes between the ligands and the enzyme’s catalytic site, corroborating the relevance of α-amylase as a possible molecular target for natural terpene compounds in vector control (da Silva et al., 2023a; Guedes et al., 2024; da Rocha et al., 2026). These observed values indicate that subtle structural modifications, such as the presence of the epoxide group present in Oxidoselin-1,3,7(11)-trien-8-one, can contribute positively to the stabilization of the ligand-protein complex, without compromising the hydrophobic fit characteristic of sesquiterpenes (da Rocha et al., 2026). Analysis of the ligand conformations, based on two-dimensional diagrams generated in BIOVIA Discovery Studio Visualizer 2025, revealed that the three compounds predominantly fit into the traditional catalytic cavity of α-amylase, interacting with residues described in the literature as fundamental for molecular recognition and catalytic activity of the enzyme (da Silva et al., 2023a). In all complexes, the recurrent participation of aromatic and hydrophobic residues was observed, such as TRP314, TRP315, TYR318, LEU412 and LEU415, which constitute the core of the active site.

**TABLE 4:** Parameters obtained from molecular docking performed on the DockThor-VS server.

Name	Score	T. Energy	I. Energy	vdW energy	Electrostatic energy
Oxidoselina-1,3,7(11)-trien-8-one	−8.880	23.338	−25.235	−20.292	−4.943
Curzerene	−8.870	25.135	−21.517	−21.076	−0.441
Selina-1,3,7(11)-trien-8-one	−8.644	29.892	−22.466	−18.180	−4.286

## 5 Conclusion

The essential oil from *E. uniflora* and its major compound–curzerene–are potential bioactive candidates for combating *Ae. aegypti*, as these substances hindered the reproduction cycle of the vector from the larval stage to oviposition. Moreover, curzerene is easily isolated from the *E. uniflora* oil by countercurrent chromatography and can be an option for use in vector control programs. This oil exhibited excellent oviposition deterrence capable of reducing oviposition by more than 73% at a concentration of 50 ppm and more than 65% at a concentration of 5 ppm. The innovation of this study was the use of the gas chromatography-electroantennography (GC-EAD) technique combined with oviposition bioassays to prove the oviposition deterrent activity of curzerene at LC_50_ (56 ppm). Our results demonstrated the potential application of the essential oil from *E. uniflora* and its major compound (curzerene) in the control of *Ae. aegypti* and provide insights for future studies on bioactive components from natural sources using a combination of analytical detection methods. Considering the growing concern with the development of resistance to synthetic products in *Aedes aegypti* larvae, natural products, such as essential oils, constitute an important source of substances that may hinder the development of resistance, as the oil is composed of different chemical products that may have synergistic effects and different modes of action, thus making resistance less likely. This aspect encourages future studies to explore the potential of bioactive agents against this mosquito.

## Patents

This work has intellectual property rights and has a national patent application for invention, utility model, certificate of addition of invention, and entry into the national phase of the PCT with the National Institute of Industrial Property (INPI) under process number BR10201901055. The patent refers to the formulation and general application of the oil as a larvicide and oviposition deterrent for *Aedes aegypti*.

## Data Availability

The original contributions presented in the study are included in the article/Supplementary Material, further inquiries can be directed to the corresponding author.
