# Supplementary material for: Larvicidal, oviposition inhibition, electrophysiological, histological, and docking activity of Eugenia uniflora L. oil against Aedes aegypti
Source: Front Pharmacol. 2026 Jun 2;17:1688998. doi: 10.3389/fphar.2026.1688998 (PMC13270230; doi:10.3389/fphar.2026.1688998)
Supplement: Supplementary file 1 [file Supplementaryfile1.docx]

Supplementary Material

Supplementary material figure S1: Maintenance cycle of the *Aedes aegypti* colony


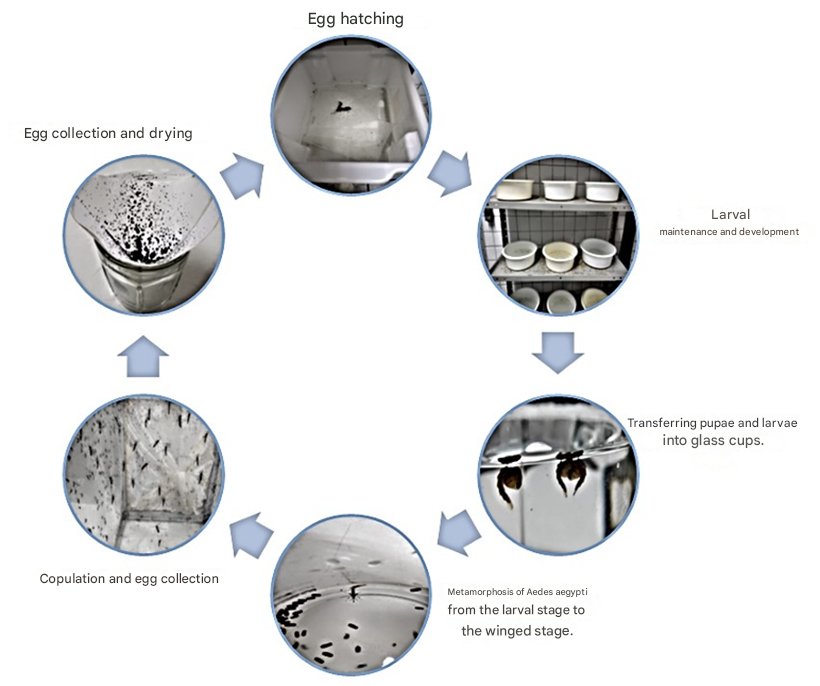


Supplementary material figure S1: Flowchart of the steps for determining the LC50


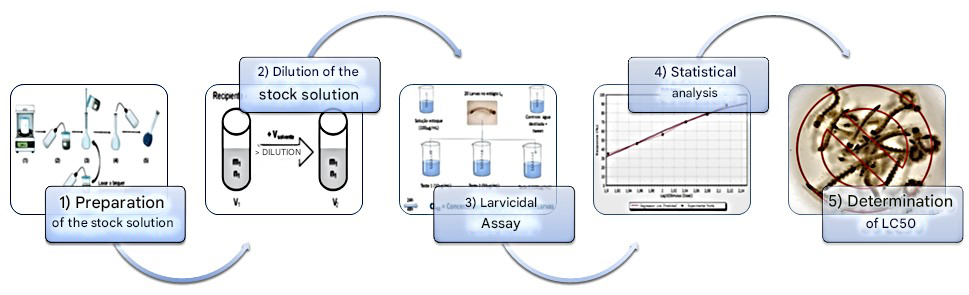


Supplementary material figure S2. Cups with 20 mL of Eugenia uniflora EO solution in different concentrations for determining the LC50 (larvicidal test)


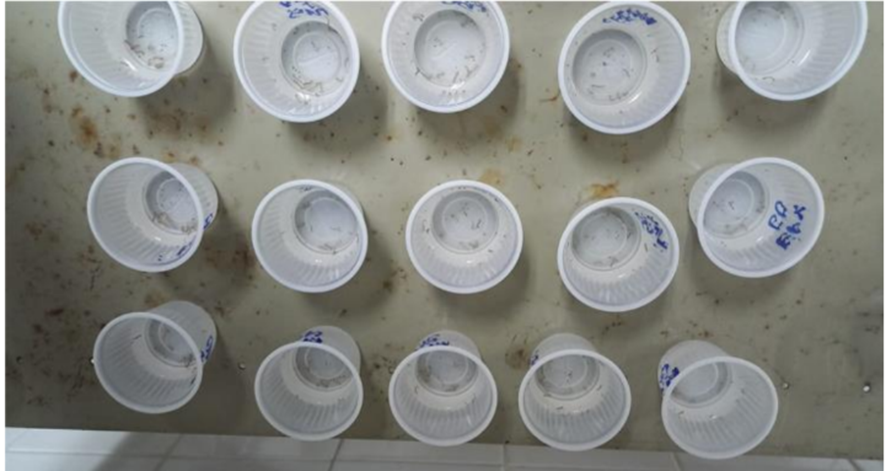


Supplementary material figure S4: Electroantennographic detector coupled to a gas chromatograph (GC-EAD).


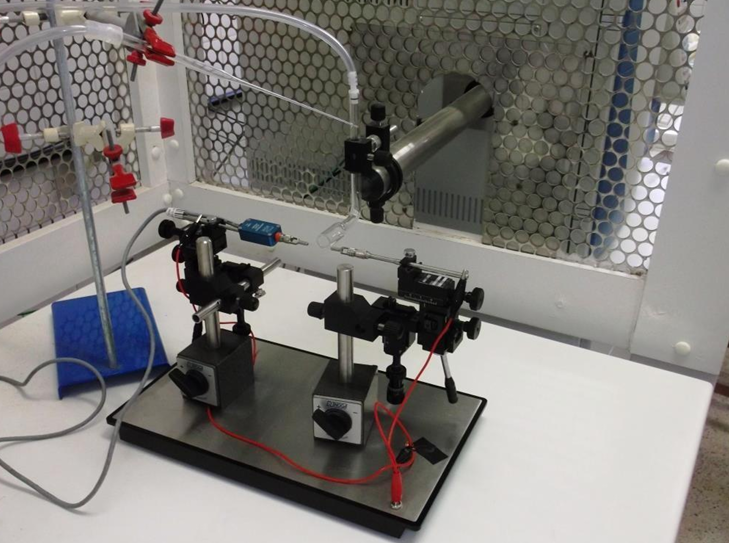


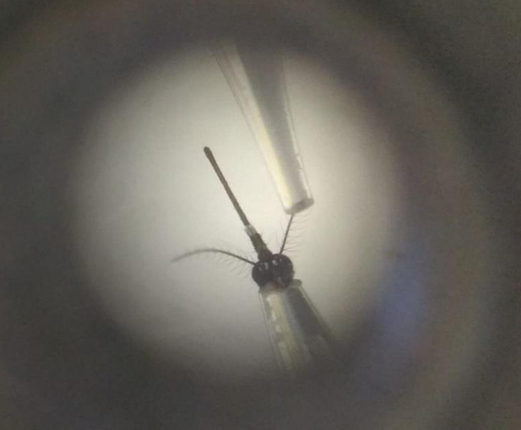


Supplementary material figure S5: Electroantennography of the head of Ae. aegypti coupled to the capillary containing the working and reference electrodes, along with Ringer's solution, to close the circuit. The system acts as a biosensor in the detection of electrophysiologically active components.


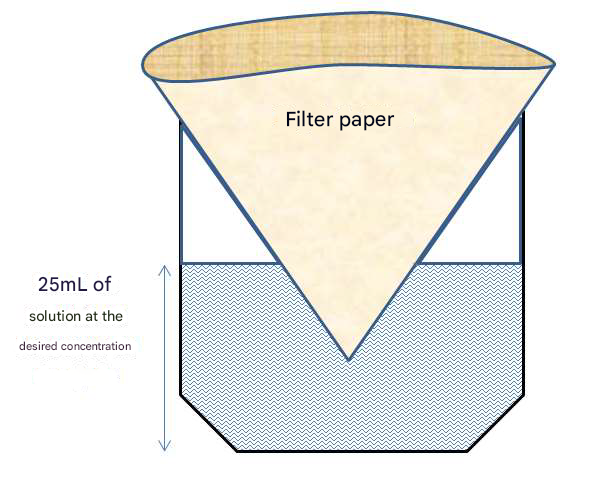

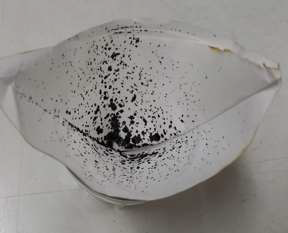


Supplementary material figure S6: Oviposition site (cup)


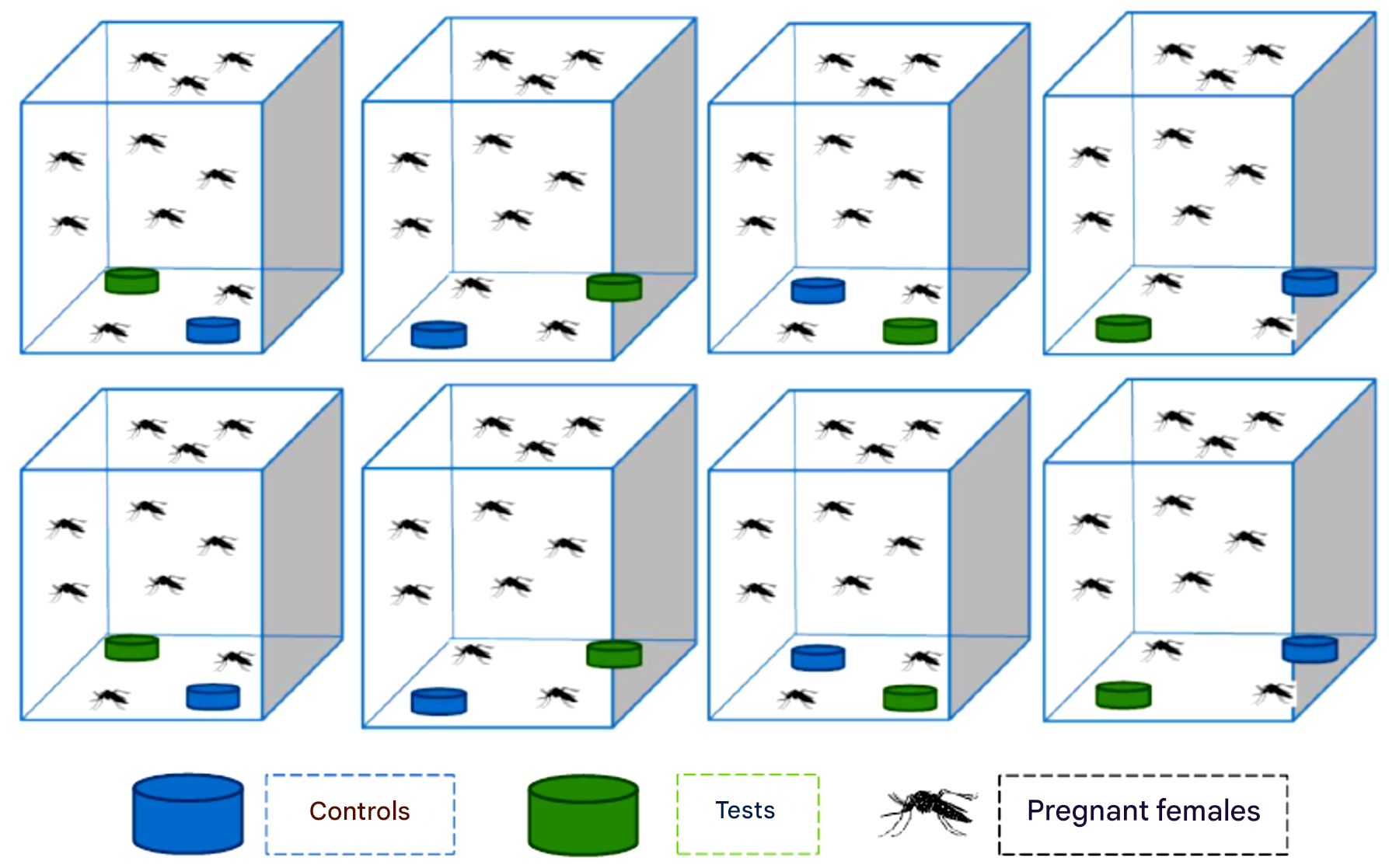


Supplementary material figure S7: Schematic of the positions of the test and control cups for the eight cages containing gravid females during the oviposition experiment


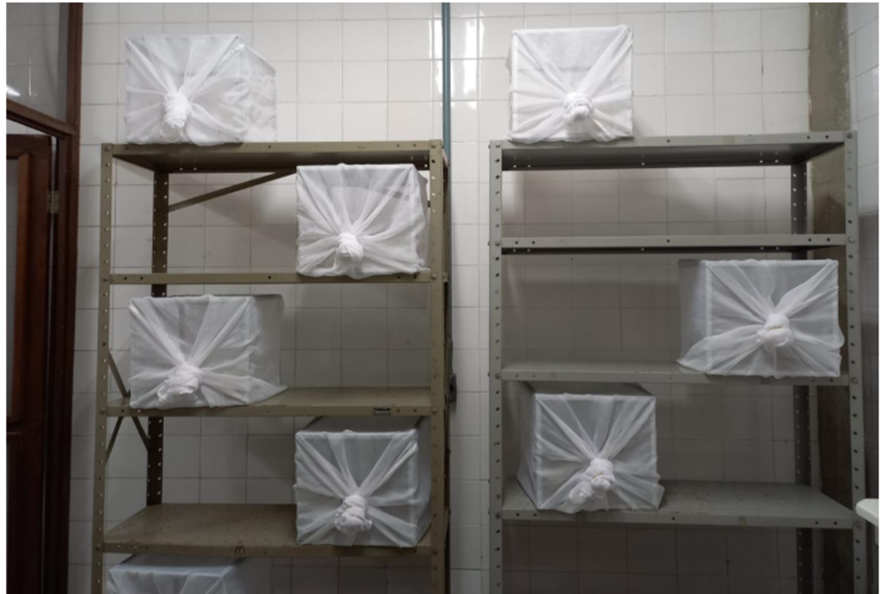


Supplementary material figure S8. Oviposition. The test is performed over a period of 16 hours in the absence of light.


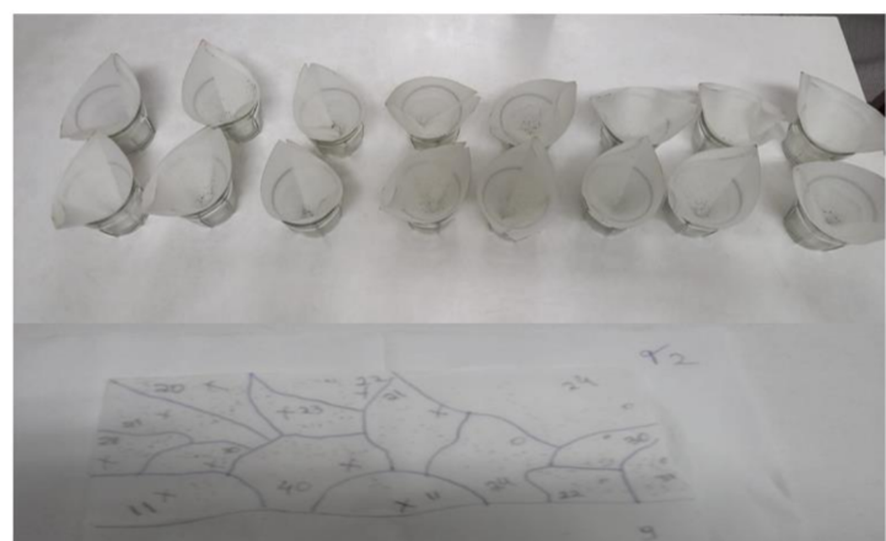


Supplementary material figure S9: Egg laying and egg count

Supplementary material figure S10: Possible results for the Oviposition Activity Index (OAI)


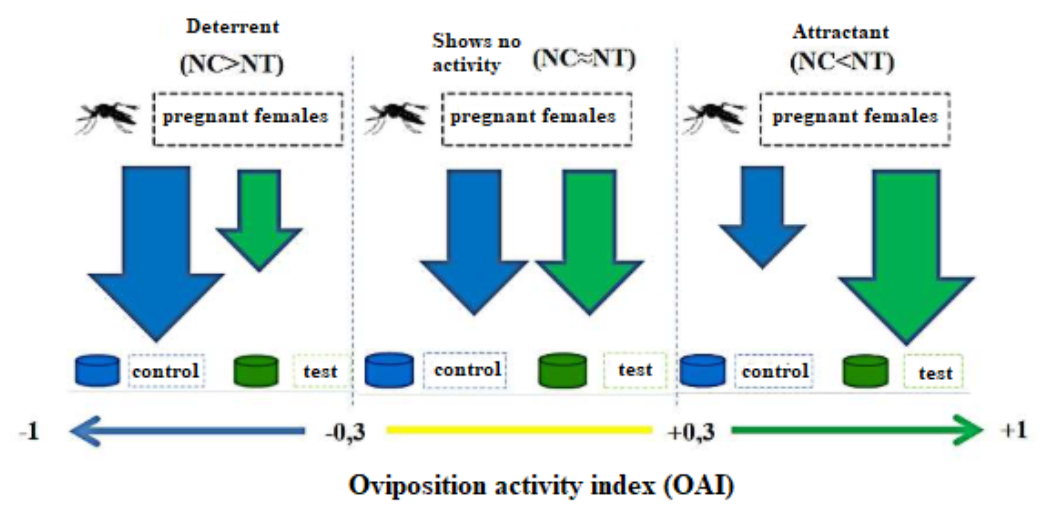


Supplementary material figure S11: chromatogram of the essential oil (EO) of *Eugenia uniflora*


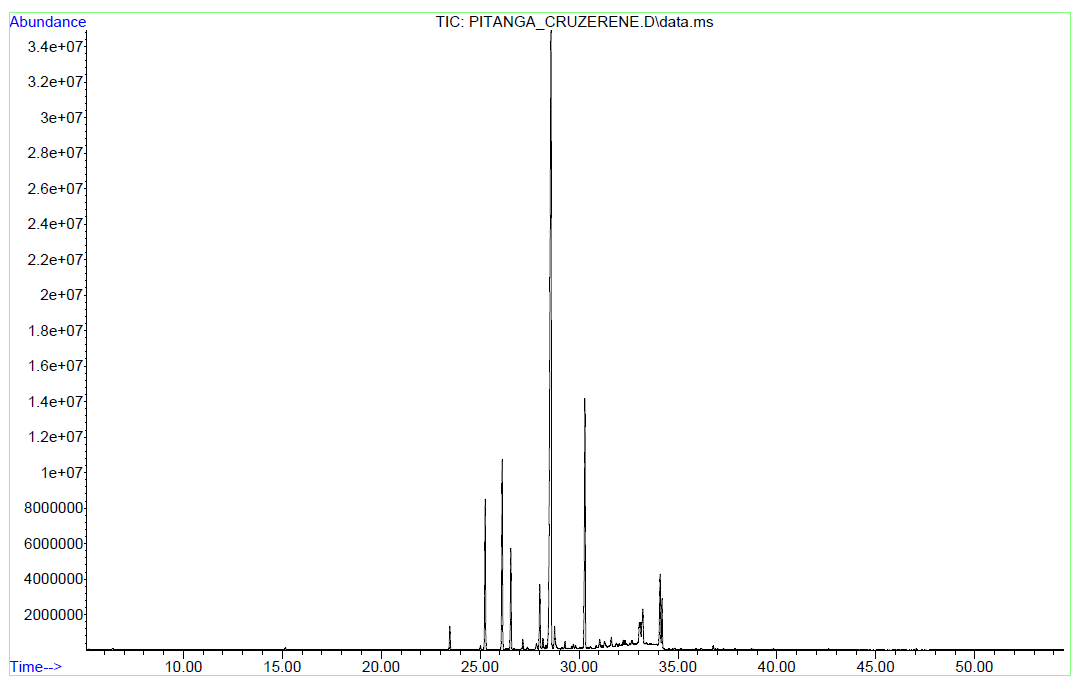


Compound (retention time) = Linalool (15.149 min); δ-Elemene (23.468 min); β-Elemene (25.254 min); (*E*)-Caryophyllene (26.116 min); γ-Elemene (26.550 min); α-Humulene (27.158min); 9-epi-(*E*)-Caryophyllene (27.385min); Germacrene D (28.015 min); Selinene β (28.170 min); Amorpha-4.7(11)-diene (28.330 min); Curzerene (28.583 min); Germacrene A (28.765min); δ-Cadinene (29.289min); Selina-3.7(11)-diene (29.821 min); Germacrene B (30.298 min); Spathulenol (30.861min); trans-β-Elemenone (31.633min); epi-α-Cadinol (32.668min); neo-Intermedeol (33.046min); Atractylone (33.226min); Germacrone (34.194 min).
